# Growth Factor Content in Human Sera Affects the Isolation of Mesangiogenic Progenitor Cells (MPCs) from Human Bone Marrow

**DOI:** 10.3389/fcell.2016.00114

**Published:** 2016-10-17

**Authors:** Marina Montali, Serena Barachini, Francesca M. Panvini, Vittoria Carnicelli, Franca Fulceri, Iacopo Petrini, Simone Pacini

**Affiliations:** ^1^Department of Clinical and Experimental Medicine, University of PisaPisa, Italy; ^2^Department of Surgical, Medical and Molecular Pathology and Critical Care Medicine, University of PisaPisa, Italy; ^3^Department of Translational Research and New Technologies in Medicine and Surgery, University of PisaPisa, Italy

**Keywords:** mesangiogenic progenitor cells, mesenchymal stromal cells, human serum, growth factors, bone marrow culture, cell-based medicinal product, *Erlotinib*, *Nintedanib*

## Abstract

Mesangiogenic Progenitor Cells (MPCs) are human bone marrow-derived multipotent cells, isolated *in vitro* under selective culture conditions and shown to retain both mesengenic and angiogenic potential. MPCs also co-isolated with multipotent stromal cells (MSCs) when bone marrow primary cultures were set up for clinical applications, using human serum (HS) in place of fetal bovine serum (FBS). MPC culture purity (over 95%) is strictly dependent on HS supplementation with significant batch-to-batch variability. In the present paper we screened different sources of commercially available pooled human AB type serum (PhABS) for their ability to promote MPC production under selective culture conditions. As the majority of “contaminating” cells in MPC cultures were represented by MSC-like cells, we hypothesized a role by differentiating agents present in the sera. Therefore, we tested a number of growth factors (hGF) and found that higher concentrations of FGF-2, EGF, PDGF-AB, and VEGF-A as well as lower concentration of IGF-1 give sub-optimal MPC recovery. Gene expression analysis of hGF receptors was also carried out both in MSCs and MPCs, suggesting that FGF-2, EGF, and PDGF-AB could act promoting MSC proliferation, while VEGF-A contribute to MSC-like cell contamination, triggering MPC differentiation. Here we demonstrated that managing hGF contents, together with applying specific receptors inhibitors (*Erlotinib*-HCl and *Nintedanib*), could significantly mitigate the batch-to-batch variability related to serum supplementation. These data represent a fundamental milestone in view of manufacturing MPC-based medicinal products.

## Introduction

Mesenchymal stromal cells (MSCs) are multipotent adult progenitor cells isolated *in vitro* from a plethora of different tissues (reviewed in Murray et al., 2014) as their perivascular origin has been demonstrated and widely accepted (da Silva Meirelles et al., 2006; Crisan et al., 2008). MSC role as skeletal tissue progenitors has been investigated in a variety of studies in animal models, and cell-based therapy approaches demonstrated their beneficial effects in the regeneration of damaged skeletal tissues including bone (Watson et al., 2014), cartilage (Richardson et al., 2015), tendons (Pacini et al., 2007), and meniscus (Yu et al., 2015). For those reasons, most of the tissue engineering strategies and regenerative medicine approaches, developed to *de novo* skeletal tissue formation, involved MSCs (Yousefi et al., 2016). Nonetheless, it has been reported that an effective tissue remodeling response is in tight correlation with the formation of vascularized tissue (Rao and Stegemann, 2013; Hutton and Grayson, 2014). Commonly, the vascularization of the newly formed tissues *in vivo* has been expected driven in large part by the host response to the implant, and trigged by hypoxic condition surrounding the grafted cells (Chamberlain et al., 2015). Nonetheless, this effect could be hypothesized in acute injuries with moderate tissue loss, but in case of large implants or poor blood supply, as in non-union fracture or pseudoarthrosis (Hak et al., 2014), the vascularization of constructs would be compromised. Modular constructs obtained applying both osteoprogenitor cells, as MSCs, and endothelial cells (ECs) or their precursors (EPCs), have been investigated as an option in order to sustain vascularization of the implants, independently from the host response (Butler and Sefton, 2012). However, MSCs have been estimated to represent around 0.001–0.01% of human bone marrow mononuclear cells (hBM-MNCs) (Subbanna, 2007; Lechanteur et al., 2016), and similarly ECs and EPCs represent rare populations in their tissues of origin (Pelosi et al., 2014), thus pre-clinical and clinical applications of these cells, alone or in combination, require extensive *ex vivo* cell expansion to obtain therapeutic cell doses. Most widely accepted MSC isolation and expansion protocols, from bone marrow, are based on basal media supplemented with fetal bovine serum (FBS) (Haynesworth et al., 1992; Prockop, 1997). Similarly, consistent fractions of FBS should be applied also in the EC and EPC isolation (Kirton and Xu, 2010). However, all supplements of animal origin expose patients to a number of risks and represent a major obstacle to comply with good manufacturing practice (GMP) guidelines (Herberts et al., 2011). The urgent need for FBS alternatives led to consider human serum (HS) as the straightforward substitute in clinical-grade MSC production (Altaie et al., 2016).

In 2009, during the attempt to produce multipotent mesenchymal stromal cells (MSCs) in xeno-free GMP-compliant culture conditions, we had shown the occurrence of mesodermal (currently renamed “mesangiogenic”) progenitor cells (MPCs), in hBM-MNC cultures when using autologous serum (AS) (Petrini et al., 2009) or pooled human AB type serum (PhABS) (Trombi et al., 2009). MPCs have been described *in vitro* as slow cycling MSC progenitors that also retain angiogenic properties (Fazzi et al., 2011). They are characterized by peculiar fried egg-shape morphology, expression of pluripotency-associated markers *OCT4* and *NANOG*, and intense nestin expression (Pacini et al., 2010). MPCs lack mesenchymal-associated markers CD73, CD90, and CD105 while showing occurrence of CD31, CD11c, and CD18.

After more than 6 years spent on MPC characterization and optimization of isolating procedures, we believe that these cells could facing a new era and possibly being involved in skeletal tissue engineering and regeneration. As MPCs demonstrated *in vitro* retaining both mesengenic and angiogenic potential, it reasonable hypothesizes the development of MPC-based implants in alternative to the modular constructs. The need of two or more different expanded cell populations giving a mesangiogenic potential to the engineered tissues could be overtaken applying cell populations like MPCs (Pacini and Petrini, 2014). However, the definition of a highly reproducible clinical-grade manufacturing process represents a first step in dealing with a possible application of MPCs in cell-based therapies. From the first report of mesangiogenic cells in human bone marrow cultures applying AS, a consistent variability in the MPC harvesting was reported. Only two out of every three samples cultured showed detectable MPC population co-isolated with MSCs, with percentages that could vary 10-fold (Petrini et al., 2009). As a consequence, we believe that the supplementation with human sera could induce considerable culture variability, during cell manufacturing process, depending on donor sex and age as well as diseases and pharmacological treatments at the time of serum collection, especially in the autologous context. Consistent production of almost pure MPC cultures has been achieved applying commercially available PhABS. Under these selective culture conditions it was possible to obtain MPCs from all the samples, with recoveries showing very low coefficient of variation (≈10%). Conversely, culturing the same samples in FBS supplemented media led to cell products constituted by MSCs only (Trombi et al., 2009). Nonetheless, we recently reported that isolation of MPCs at high grade of purity (over 95%) would be possible only after accurate screening of commercially available PhABS (Montali et al., 2016). In fact, a small number of tested batches produced cell products with consistent percentages of MSC-like cell population, compromising the purity of the MPC preparations. In order to define specific sera properties, allowing the optimal MPC manufacturing process, would be helpful the comparisons between PhABS with good performances and PhABS promoting MSC “contamination.”

Human serum contains a large and untreatable number of biological active molecules possible affecting the MPC isolation and inducing MSC proliferation (Rodrigues et al., 2010). However, recent efforts in the establishment of serum-free media for MSC *in vitro* expansion, demonstrated that human platelet lysate (hPL) could efficiently replace serum in culture (reviewed in Burnouf et al., 2016). This important finding narrows the choice of essential components to platelet granules content (Fekete et al., 2014). Platelets contain a lot of potent biological active molecules (Semple et al., 2011), mainly stored in the α-granules (Golebiewska and Poole, 2015), in addition to important factors involved in coagulation. These molecules include different chemokines (Semple et al., 2011) and human growth factors (hGFs), such as epidermal growth factor (EGF), basic fibroblast growth factor (FGF-2), platelet derived growth factor isoforms (PDGF-AA, -AB, and-BB), insulin-like growth factor-1 (IGF-1), vascular endothelial growth factor (VEGF), and some others (Nurden et al., 2008).

In order to define reproducible MPC manufacturing process, in the present paper we moved a first step focusing on the effect of the platelet-derived hGFs, mentioned above, on the MPC production. EGF, FGF-2, PDGFs, IGF-1, and VEGF have been reported regulating proliferation and survival of BM-derived MSCs. Thus, we hypothesize that these five hGFs or some of them could promote expansion of MSCs, during primary bone marrow cultures intended for MPC production. Here we compare six different commercial PhABS, selected on the basis of their different performance, attempting to correlate the percentage of “contaminant” MSC-like cells with the serum concentration of those hGFs.

## Materials and methods

### Screening of human sera for MPC isolation

The study has been performed according to the declaration of Helsinki and the sample collection protocol was approved by the ethical committee of “*Azienda Ospedaliero-Universitaria Pisana*”. Bone marrow aspirates were obtained after written consent from 8 patients (4M/4F, median age 65), undergoing orthopedic surgery for hip replacement. Soon after femoral neck osteotomy, approximately 10 ml of bone marrow were aspirated, using a 20 ml syringe containing 500 U.I. of heparin, and promptly sent to the cell culture facility. Samples were diluted 1:4 and hBM-MNCs collected by density gradient centrifugation using Ficoll-Paque^TM^PREMIUM (GE Healthcare, Uppsala, Sweden). After two washes in Dulbecco's Modified Phosphate Buffer (D-PBS, LifeTechnologies, Carlsbad, USA-CA) cells were plated at 8 × 10^5^/cm^2^ in hydrophobic six-well plates for suspension culture (Greiner Bio-One, Kremsmünster, Austria) and cultured in minimal essential medium supplemented with PhABS as previously described (Montali et al., 2016). Briefly, low-glucose Dulbecco's modified Eagle medium (DMEM, LifeTechnologies) was supplemented with 2 mM Glutamax® (LifeTechnologies), 100 μg/mL gentamicin (LifeTechnologies), and 10% PhABS. We tested six different commercially available PhABS (AB type and off-the-clot) from four different manufacturers (Table 1). Culture medium was changed every 48 h. After 7–8 days, plates were morphologically screened for MPCs using an inverted microscope, and cells subsequently detached by TrypLE® Select (LifeTechnologies) digestion and washed in D-PBS.

**Table 1:** **PhABS details**.

**Code**	**Lot#**	**Manufacturer**	**Type**	**Production method**	**Gender**	**Origin**
LZM	N.R.^*^	Lonza, Basel, Switzerland	AB	Off-the-clot	Male only	USA
LZMF	N.R.^*^	Lonza, Basel, Switzerland	AB	Off-the-clot	Not declared	USA
SERL	E8051213	SeraLab, West Sussex, UK	AB	Off-the-clot	Male only	USA
SIGM	SLBF-3954V	Sigma Aldrich, St. Luis, USA	AB	Off-the-clot	Not declared	USA
BIOW1	S10443S4190	BioWest, Nuaillé, France	AB	Off-the-clot	Male only	EU
BIOW2	S10169S4190	BioWest, Nuaillé, France	AB	Off-the-clot	Male only	EU

* *N.R., Not Recorded*.

### MSC cell culture

Duplicate hBM-MNC samples collected by density gradient centrifugation were plated at 2 × 10^5^/cm^2^ in gas-treated culture plates for adherent cells. DMEM was supplemented with 2 mM Glutamax® (LifeTechnologies), 100 μg/mL gentamicin (LifeTechnologies), and 10% FBS (LifeTechnologies). Culture medium was changed after 48 h to remove non-adherent cells and refreshed twice a week.

### Flow cytometry

To identify MPCs and MSC-like cells (Keating, 2012) in MPC primary cultures freshly detached cells were incubated with anti-CD90 FITC-conjugated, anti-CD73 PE-conjugated, anti-CD31 PE/Cy7-conjugated, anti-CD18 APC-conjugated, and anti-CD45 VioBlue® -conjugated (Miltenyi Biotec, BergischGladbach, Germany) antibodies for 30′ at 4°C in the dark and washed twice in MACSQuant® Running Buffer (Miltenyi Biotec). Data were acquired using MACSQuant® flow cytometer and analyzed by MACSQuantify® Analysis Software (MiltenyiBiotec). MPCs were identified as CD31^+^CD18^+^CD45^low^CD73^neg^CD90^neg^ events and MSCs as CD31^neg^CD18^neg^CD45^neg^CD73^bright^CD90^bright^ events. Statistical analysis was performed by one-way analysis of variance (ANOVA) test and Dunnett's post-test for multiple comparison. Results were expressed as mean value ± standard error (SE).

### Gene expression

MPCs and MSCs from primary cultures were washed twice in D-PBS, and pellets cryo-preserved in liquid nitrogen to be processed. Total RNA extraction was performed soon after thawing, using RNeasyMicro Kit (Qiage, Hilden, Germany) according to manufacturer. RNA samples (100 ng) were retro-transcribed using QuantiTect® Reverse Transcription Kit (Qiagen) and 2 μl samples of 10-fold cDNA dilutions were amplified by quantitative Real Time PCR (qRT-PCR), using iCycler-iQ5 Optical System (Bio-Rad, Hercules, USA-CA) and SsoAdvancedSYBR Green SuperMix (Bio-Rad). Samples were run in duplicate. Primer pairs (Sigma-Aldrich, St. Luis, USA-MO) were designed to detect growth factor receptor genes: *BMPR1A, BMPR2, EGFR, FGFR1, FGFR2, FGFR3, IGF1R, IGF2R, KDR, PDGFRA, PDGFRB, TGFBR1, TGFBR2*, and *TGFBR3* (Supplementary Table S1). Relative quantitative analysis was performed following 2^−ΔΔCt^ Livak method (Livak and Schmittgen, 2001). Normalization was performed by using *RPL13A* and *ACTB* housekeeping genes. Values were reported as log-ratios of mean MPC/MSC normalized fold expression.

### Enzyme-linked immunosorbent assay (ELISA)

Growth factor quantification was performed on 200 μl aliquots of serum batches, by colorimetric solid phase ELISA. In particular; epidermal growth factor (EGF), fibroblast growth factor-2 (FGF-2), and platelet-derived growth factor AB (PDGF-AB) were quantified using Quantikine® immunoassay kit (R&D Systems, Minneapolis, USA-MN). Vascular endothelial growth factor A (VEGF-A) was quantified by VEGF-A (human) BioLISA Kit Bender MedSystems® (Vienna, Austria) and insulin-like growth factor-1 (IGF-1) by IGF-1 600 ELISA kit from DRG International (Marburg, Germany). Assays were performed according to manufacturer. Percentages of MSC-like cells in MPC primary cultures were correlated with growth factor concentrations in specific sera batches by Spearman's correlation.

### Recombinant hGF treatments during MPC isolation

hBM-MNCs were obtained from 4 patients (2M/2F, median age 68) and processed for MPC isolation as described above, applying two different sera batches: LZM, already screened for good performance and BIOW2 that produced cultures with consistent MSC-like cell “contamination.” LZM cultures were also performed in presence of 50 ng/ml rhEGF (ThermoFisher Scientific, Waltham, MA USA), 20 ng/ml rhFGF-2 (ThermoFisher Scientific), 50 ng/ml rhPDGF-AB (PeproTech EC Ltd., London, UK) or 50 ng/ml rhVEGF (ThermoFisher). Combined treatments were also performed adding rhEGF and rhFGF-2, or rhVEGF and rhPDGF-AB. In parallel BIOW2 cultures were also supplemented with 100 ng/ml rhIGF-1 (PeproTech EC Ltd., London, UK), 30 ng/ml rhIGF-2 (PeproTech EC Ltd.) alone or in combination. The culture media were changed every 2 days restoring concentrations of the hGFs. After 7 days cells were detached by TrypLE® Select digestion and processed for flow cytometry to quantify MPC and MSC percentages in the cultures, as described above. Data were collected in duplicate and reported as median values ± standard error (SE). One-way ANOVA test, coupled with Dunnett's post-test, was applied to identify significant differences.

### Inhibition of hGF receptors, during primary cultures

Further six bone marrow samples (4M/2F median age 68) were processed, as described above, to obtain MPC culture in BIOW2 sera batch with or without adding 4 nM of *Erlotinib*-HCl (OSI-744, SelleckChem, Houston, USA-TX), a potent EGFR inhibitor, and 100 nM of *Nintedanib* (BIBF 1120, SelleckChem) to simultaneously inhibit VEGF, FGF, and PDGF receptors. Cell cultures were performed in duplicate in six-well culture plates for suspension cultures, media were changed after 48 and 72 h restoring inhibitors concentration and at day 6 cultures were detached for flow cytometry analysis to quantify mesenchymal CD73^+^CD90^+^ population. Similarly and in parallel, MPC cultures were performed applying LZM sera batch in presence or absence of 50 nM of *Linsitinib* (OSI-906, SelleckChem) as IGF-1R inhibitor. Significant differences were revealed by one-way ANOVA test, and the inhibition index was calculated as difference in CD73^+^CD90^+^ percentages in treated and no-treated cultures divided by the percentage in the non-treated. Data were collected in duplicate and reported as median values ± standard error (SE).

### Inhibition of hGF receptors, during mesengenic differentiation of MPCs

*Erlotinib*-HCl and *Nintedanib* were also applied during mesengenic differentiation of MPCs. Briefly, MPC culture in LZM sera batch were validated for a purity higher than 95% and re-plated at 20,000 cells/cm^2^ in gas-treated 6-well plates for adherent cultures. After overnight incubation medium was replaced with StemMACS^TM^ MSC expansion XF medium (Miltenyi Biotec), according to previously reported protocol (Montali et al., 2016). In parallel, mesengenic differentiation was performed in presence of 4 nM of *Erlotinib*-HCl and 100 nM of *Nintedanib*. Cultures were maintained changing medium twice a week and restoring inhibitor concentration. After 7 days mesengenic differentiation was evaluated by AlamarBlue® reduction assay (LifeTechnologies) as previously reported (Fazzi et al., 2011).

## Results

**Figure 1:**
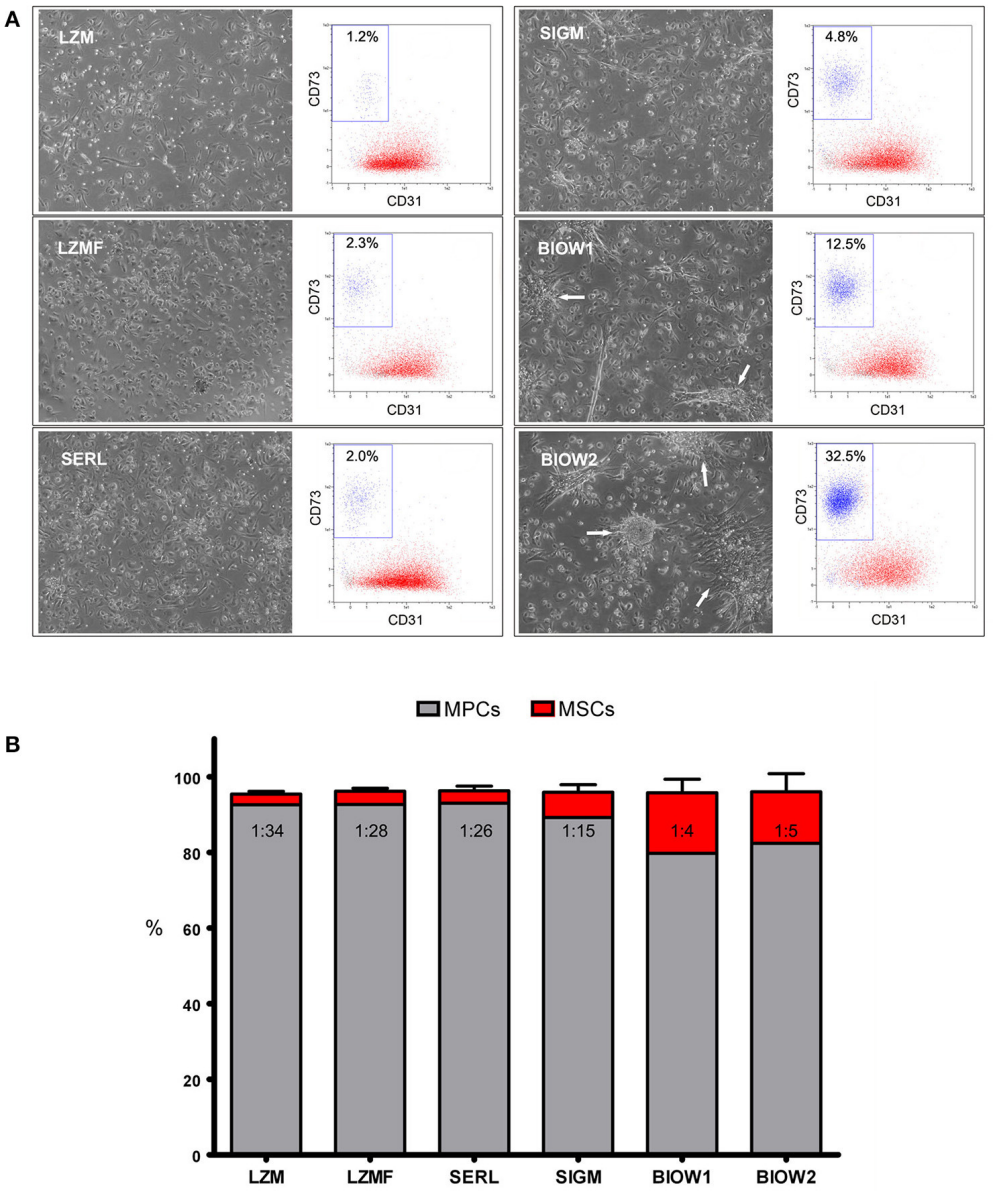
**Analysis of different PhABS in MPC cultures from bone marrow. (A)** Six different PhABS were tested for their efficiency in the isolation of MPCs. After 6 days of culture, LZM, LZMF, and SERL originated MPC cultures at high grade of purity. Cultures supplemented with SIGM showed rare MSC-like cells, while BIOW1 and BIOW2 produced a number of MSC-like cells usually clustered in proliferating foci or in rounded colonies (white arrows). Purity of cultures was estimated by flow cytometry. CD73^bright^CD90^bright^CD31^neg^ (blue dots) percentages lower than 5.0% accounted for a good performance in MPC (red dots) production. **(B)** Mean MSC/MPC ratio in LZM, LZMF, and SERL varied from 1:26 to 1:34, as opposed to 1:4 and 1:5 detected in SIGM, BIOW1, and BIOW2.

We were able to isolate and grow MPCs from hBM-MNC primary cultures supplemented with all six PhABS under test. Cell yields were adequate (1.20 ± 0.23 × 10^5^ cells/well, *n* = 8) to perform further analysis. Cultures supplemented with *Lonza* PhABS from males (LZM) or from males and females (LZMF) showed to be constituted almost exclusively by cells with the typical fried egg-shape morphology that identifies MPCs (Figure 1A; Petrini et al., 2009). Flow cytometry confirmed the characteristic CD31^+^CD18^+^CD45^low^CD73^neg^CD90^neg^ MPC phenotype for over 95% of the cell population (red dots in Figure 1A) beside a very small CD31^neg^CD18^neg^CD45^neg^CD73^bright^CD90^bright^ population of MSC-like cells (blue dots in Figure 1A; Montali et al., 2016). Similar results were obtained using *SeraLab* PhABS (SERL) (Figure 1A). *Sigma-Aldrich* PhABS (SIGM) supplementation resulted in the presence of occasional spindle-shaped MSC-like cells together with a mild increase in the percentage of MSC immunophenotype (Figure 1A). Cultures supplemented with two lots of *BioWest* PhABS (BIOW1, BIOW2) showed a large population (10–30%) of MSC-like cells, co-isolated with MPCs. Most MSC-like cells were organized in clusters or in colonies (white arrows in Figure 1A).

We previously defined the cut point for MPC cell production as 95% of CD31^+^CD18^+^CD45^low^CD73^neg^CD90^neg^ cells within MPC primary cultures (Montali et al., 2016). Three out of the six serum batches resulted suitable for MPC production, showing percentages of MSC-like cells lower than 5% with no significant variation: LZM (2.7 ± 0.6%), LZMF (3.3 ± 0.5%), SERL (3.3 ± 1.2%). SIGM was borderline (5.9 ± 1.4%), while BIOW1 and BIOW2 turned out not to be fitting because of significantly (*p* < 0.01 to LZM, *p* < 0.05 to LZMF and SERL) higher percentages of MSC-like cells (17.7 ± 4.8% and 16.2 ± 3.9%, respectively) resulting in MSC/MPC frequencies around 1:4–1:5 (Figure 1B).

**Figure 2:**
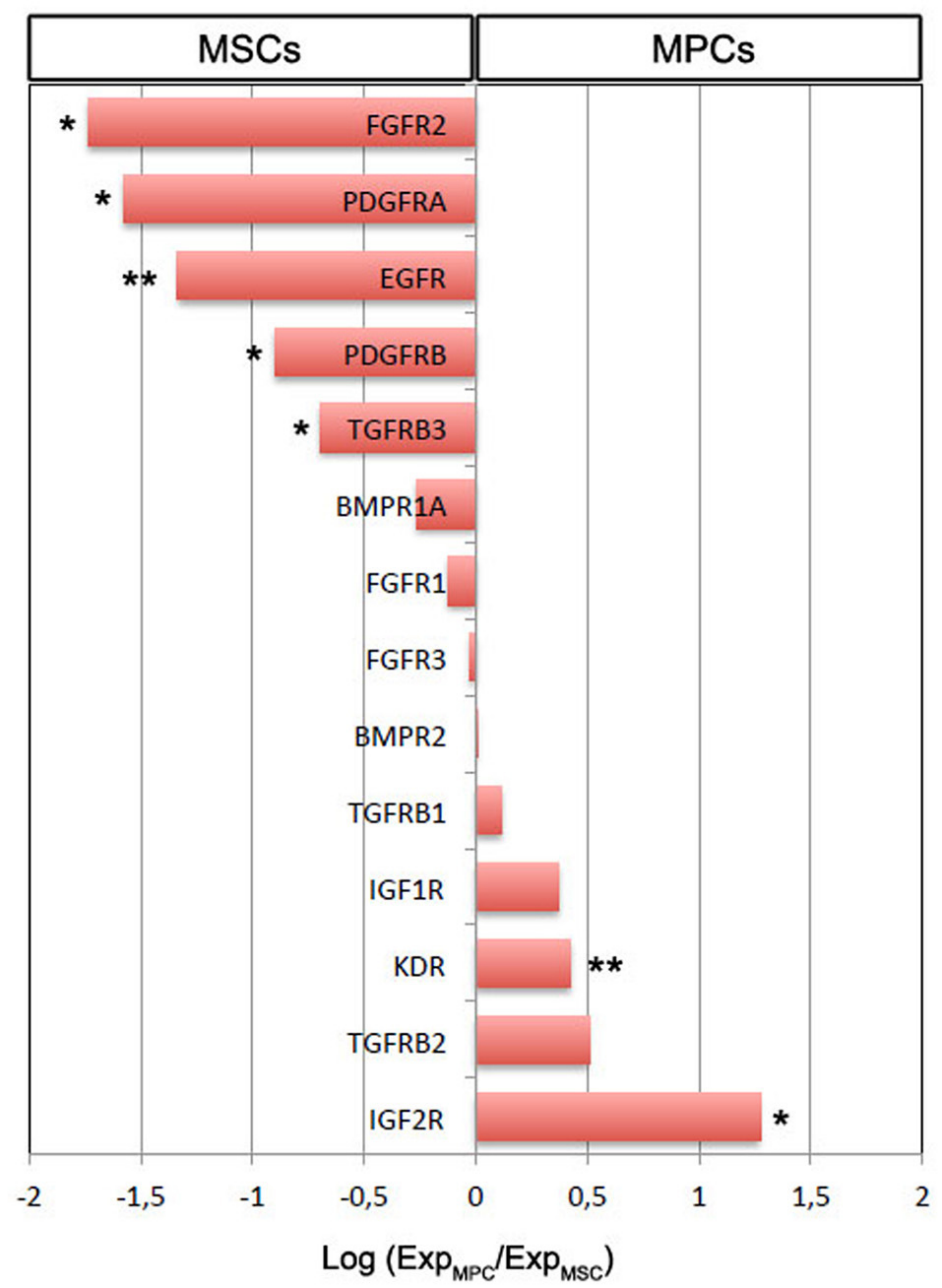
**Gene expression analysis of growth factor receptor genes in MSCs vs. MPCs**. The majority of the receptor genes resulted differentially expressed (^*^p < 0.05, ^**^p < 0.01). Expression of *FGFR2, PDGFRA*, and *EGFR* was over one log higher in MSCs. Conversely, 10-fold higher *IGF2R* expression was evidenced in MPCs.

MPCs from LZM supplemented cultures were selectively chosen for gene expression analysis of growth factor receptors in comparison to standard FBS-cultured MSCs. Expression of 3 out of the 14 genes analyzed was over one log higher in MSCs: *FGFR2* (−1.74 log, *p* < 0.05, *n* = 8), *PDGFRA* (−1.58 log, *p* < 0.05, *n* = 8), and *EGFR* (−1.33 log, *p* < 0.01, *n* = 8). *PDGFRB* and *TGFR3* were also significantly more expressed in MSCs although at lower levels (−0.90 and −0.69 log, respectively, *p* < 0.05, *n* = 8). Conversely, *IGF2R* and *KDR* expression was significantly higher in MPCs, with *IGF2R* only over one log higher (1.27 log, *p* < 0.01, *n* = 8, Figure 2). Higher levels of *IGF1R* and *TGFRB2* were detected in MPCs with a confidence limit lower than 90% (0.37 log, *p* = 0.373 and 0.51 log, *p* = 0.198, *n* = 8, respectively). *BMPR1A, BMPR2, FGFR1, FGFR3*, and *TGFRB1* showed no significant differences.

**Figure 3:**
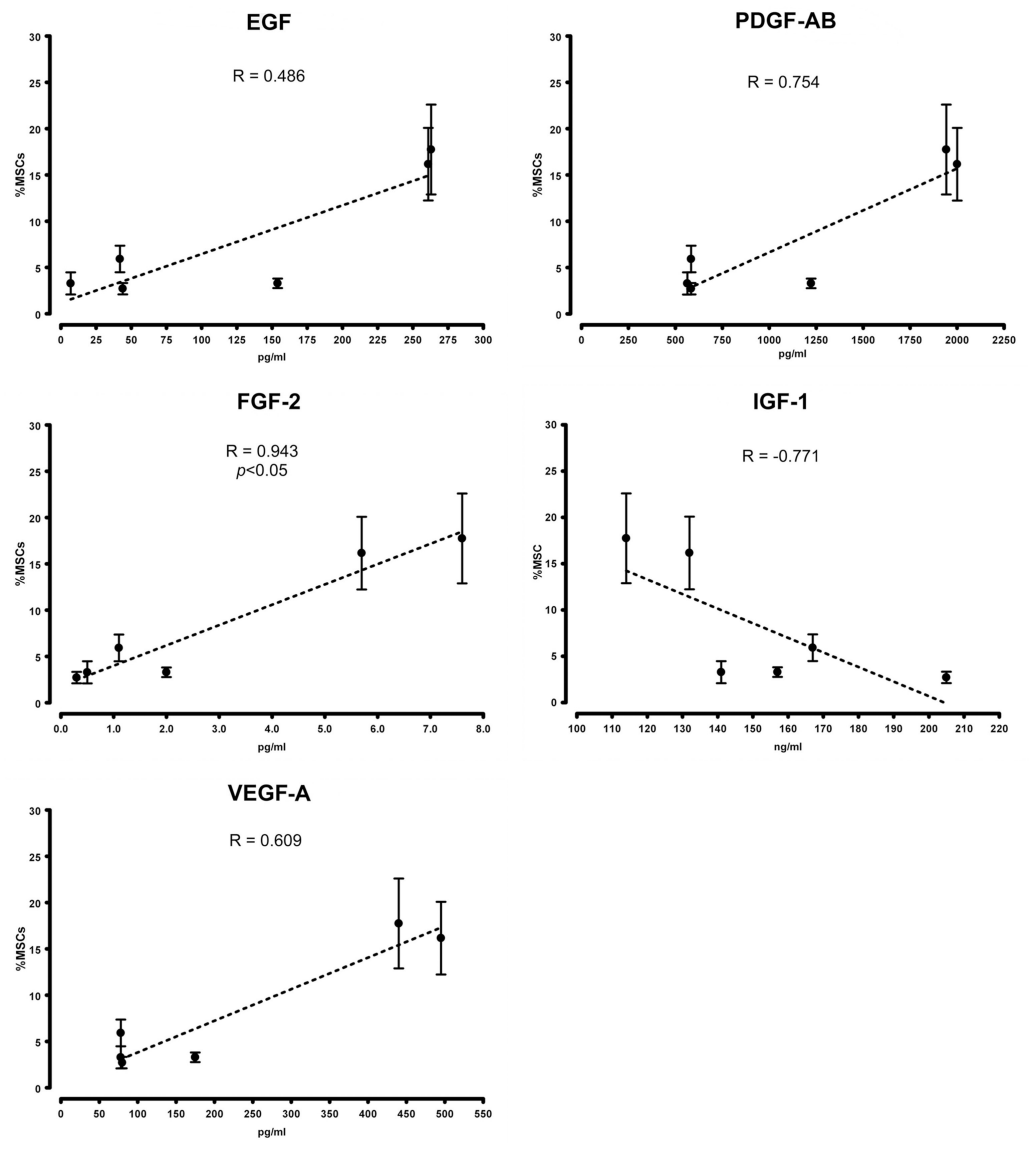
**Growth factor serum content correlated with cell composition in primary cultures**. Higher concentrations of EGF, FGF-2, PDGF-AB, and VEGF-A were detected in PhABS that gave origin to cultures with higher percentages of “contaminating” MSC-like cells. Good performance in the initiation of MPC cultures at high grade of purity correlated with elevated levels of IGF-1.

**Figure 4:**
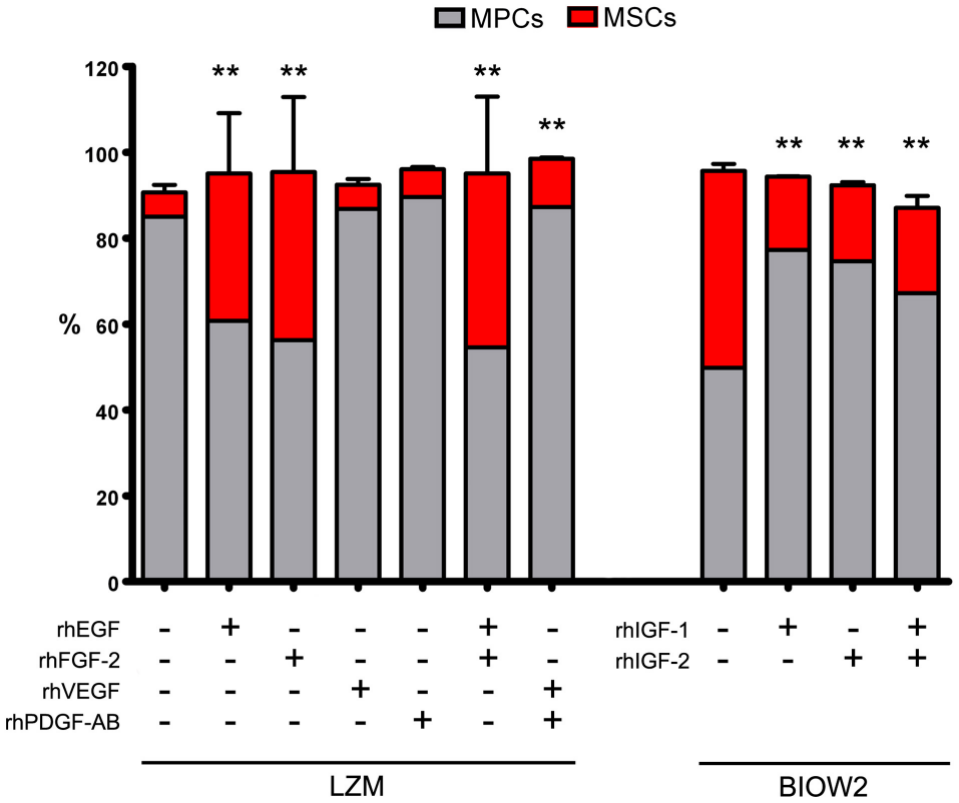
**Managing hGF medium contents, by adding recombinant factors, affected the sera performance during MPC isolation**. Adding rhEGF or rhFGF-2, alone or in combination, to the cultures supplemented with good performance serum (LZM), resulted in significantly increased MSC-like population (red bars). Under these conditions, MPC/MSC ratio was comparable to those reported applying MSC-inducing PhABS. Treating with rhVEGF or rhPDGF-AB had no effects on MPC isolation, but a combination of those two hGFs produced mild but significant increase in MSC-like counterpart. BIOW2 batch was selected as PhABS with poor performance, evaluating the effects of rhIGF-1 and rhIGF-2 during MPC isolation. The treatment with rhIGFs, either alone or in combination, produced a 50% reduction of the MSC-like population (^**^p < 0.01, respect to control).

The “contamination” of MSC-like cells in MPC primary cultures was correlated to the concentration of growth factors in the different sera. Higher concentrations of EGF, FGF-2, PDGF-AB and VEGF-A were consistently found in BIOW1 and BIOW2 that produced cultures with higher percentages of MSC-like cells (Table 2). FGF-2 revealed a dose-dependent correlation showing a Spearman ratio of 0.943 (*p* < 0.05, *n* = 8, Figure 3). In order to confirm the involvement of these hGFs in the poor performance of a pooled sera batch, we separately added consistent amount these factors to LZM supplemented cultures. Percentages of MSC-like cells resulted significantly increased in the rhEGF (34.3 ± 14.0%, *p* < 0.01, *n* = 4) and rhFGF-2 (39.2 ± 17.5%, *p* < 0.01, *n* = 4) treated primary cultures, respect to LZM control (4.6 ± 1.7%, *n* = 4, Figure 4). Combining these two hGFs produced similar results (40.5 ± 17.9%, *n* = 4), with no significant differences respect to cultures treated with EGF or FGF-2 separately. Conversely, adding rhVEGF (5.6 ± 1.4%, *n* = 4) or rhPDGF-AB (6.4 ± 0.5%, *n* = 4) resulted having no significant effects on MPC cultures when added separately. Combination of those hGF instead showed a mild increase in MSC-like cell percentages (11.3 ± 0.3%, *p* < 0.01, *n* = 4), resulting in cultures with purity lower than the cut-off point established for feasible MPC products.

**Table 2:** **PhABS growth factor concentrations**.

**PhABS batch**	**EGF (pg/ml)**	**FGF-2 (pg/ml)**	**PDGF-AB (pg/ml)**	**VEGF-A (pg/ml)**	**IGF-1 (ng/ml)**
LZM	44	0.3	584	80	205
LZMF	154	2.0	1223	175	157
SERL	7	0.5	564	78	141
SIGM	42	1.1	584	78	167
BIOW1	263	7.6	1943	440	114
BIOW2	261	5.7	2000	495	132

Medium supplemented with BIOW2 was selected in order to verify if higher concentration of IGFs could be related to the good performances of feasible sera batches. A consistent reduction, around 50%, of the MSC “contamination” was reported in rhIGF-1 (17.1 ± 0.1%, *p* < 0.01, *n* = 4) and rhIGF-2 (17.7 ± 0.2%, *p* < 0.01, *n* = 4) treated cultures, alone or in combination (19.8 ± 2.8%, *p* < 0.01, *n* = 4) respect to BIOW2 control (45.8 ± 1.6%, *n* = 4, Figure 4).

**Figure 5:**
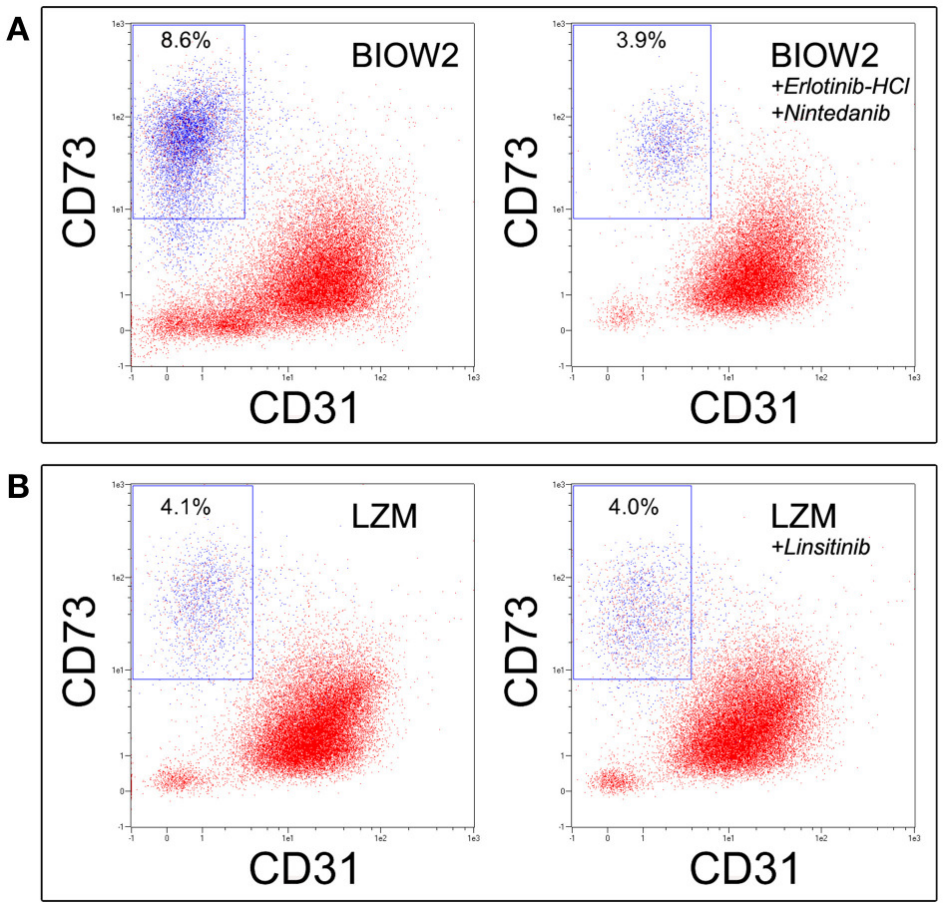
*****Erlotinib***-HCl in combination with ***Nintedanib*** allowed recovering quality MPC products applying sera with poor performance. (A)** The simultaneous inhibition of EGFR, FGFRs, PDGFRs and VEGFRs during culture of hBM-MNCs in BIOW2 serum batch, produce a 50% reduction in the CD73^+^CD90^+^ population, leading to a cell product comparable to once produced in LZM. **(B)** Conversely, the inhibition of IGF-1R exerted by *Linsitinib* did not affect the MPC production.

The effect of high concentration of EGF, FGF-2, VEGF and PDGFs in the poor performance of pooled sera batches, during MPC production, was also confirmed by receptor inhibition experiments. In fact, the addition of *Erlotinib*-HCl and *Nintedanib* to BIOW2 cultures resulted in the 50% reduction of the CD73^+^CD90^+^ population (mean inhibition index: 54.2 ± 7.7%, *p* < 0.05, *n* = 6) at the primary cultures, producing suitable MPC products (>95% of purity) comparable to ones obtained applying LZM (Figure 5A). Elevated levels of IGF-1 were instead detected in the sera batches with low mesenchymal contamination (LZM, LZMF) but the inhibition of its receptors did not produce any detectable effect during primary culture (Figure 5B).

*Erlotinib*-HCl and *Nintenadib* also affected the *in vitro* MPC mesengenic differentiation, with a decreased AlamarBlue® reduction from 20 to 40% respect to control (mean inhibition index: 22.7 ± 10.9%, *p* < 0.05, *n* = 6). Conversely, *Linsitinib* produced no detectable effects (data not shown).

## Discussion

*Ex vivo* expansion of bone marrow-derived multipotent cells requires supplementation of basal culture medium in order to sustain cell proliferation while maintaining cell differentiation capability (Bernardo et al., 2011). FBS-based expansion protocols have been used in the very first clinical applications of these promising cells (Le Blanc et al., 2004, 2008). However, safety concerns have been raised regarding use of supplements of animal origin in clinical-grade expansion protocols (Sensebé and Bourin, 2008; Reinhardt et al., 2011; Sensebé et al., 2011). Consequently, many efforts have been made to replace animal derived supplements and reagents with human blood derivatives as autologous serum (AS) (Stute et al., 2004), pooled human AB serum (PhABS) (Bieback et al., 2009), platelet lysate (PL) (Hemeda et al., 2014) or a combination of them (Kocaoemer et al., 2007; Muraglia et al., 2015).

In this view, during the last years our group has been focusing on the definition of a clinical grade protocol to culture bone marrow-derived multipotent cells by replacing FBS with AS or PhABS. Our efforts led to the first description of *mesangiogenic progenitor cells* (MPCs) as previously not described cell population, co-isolated in AS-supplemented MSC cultures (Petrini et al., 2009). In parallel, a group of colleagues demonstrated the long-term efficacy and safety of this kind of cell products for the healing of atrophic pseudarthrosis of the upper limb (Giannotti et al., 2013). In this paper, Authors also hypothesized that MPCs detected in the applied cell populations, even if at low percentage (1–10%), could contribute to the long lasting healing (follow-up at 76 months) reported for the eight enrolled patients. This interesting hypothesis could be corroborated by the idea that implanted MPCs could provide efficient tissue regeneration, differentiating into *early* MSCs, as well as contribute to the vascularization of the engineered construct due to their demonstrated angiogenic properties (Fazzi et al., 2011). After these encouraging clinical results, we believe that manufacturing MPC-based medicinal products could provide a new promising tool in skeletal tissue engineering.

As MPCs have been reported being not-proliferating cells, the expansion is not actually permitted. Nonetheless, the frequency of MPCs has been estimated around 1% of hBM-MNCs, which is hundred to thousand times higher respect to MSCs (Trombi et al., 2009), leading us to hypothesized cell-based therapies involving this non-expanded multipotent progenitors. The possible application of not *in vitro* amplified cell products carries significant advantages in terms of: (i) a reduced risk of cell transformation, (ii) reduced cellular senescence, and (iii) reduced exposition to bacterial and viral contamination, minimizing the culture time (Schneider et al., 2010). However, this could provide limited number of cell doses forcing to investigate any possible clinical application of MPCs in the context of personalized cell therapies, which involved small-scale CBMP production similar to that described by Giannotti et al. (2013). Anyway, in the attempt to define manufacturing conditions, selective for MPCs, we observed large variability in MPC yield using AS as well as different manufacturers and batches of commercial PhABS.

In general, CBMPs represent complex biological products displaying high rate of intrinsic variability, mainly derived from two sources: (i) process starting material and (ii) process conditions (Williams et al., 2012). In particular for MPC-based product, the first mentioned source of variability could not be eliminated in the autologous context where starting materials are obtained by different patients. Moreover, even if the variability related to the production process could be significantly reduced applying automated and well-controlled manufacturing process (Liu et al., 2010; Pacini, 2014), the serum supplementation represents an inescapable source of variability. Here we showed such variability to correlate with some of the most important growth factors contained in platelet lysates (Fekete et al., 2012), whose concentrations in the serum fluctuate regardless of donors' age, sex or production method. Best performance in MPC production was obtained with serum concentrations of IGF-1 over 150 ng/ml combined to low levels of EGF (<160 pg/ml), FGF-2 (<2.0 pg/ml), PDGF-AB (<1200 pg/ml), and VEGF-A (<180 pg/ml). Screening these five growth factors could provide a simple means to select high quality HS batches for the production of MPC-based CBMPs. Moreover, data presented here also provide interesting application for *Erlotinib*-HCl and *Nintedanib* in the production of MPC in autologous serum, where any possible source of variability, related to fluctuations in hGF serum concentration, could be mitigated by receptors inhibition. This could possibly results in qualified MPC products independently by patient sex, age, etc.

Taking in consideration only hGFs as serum factors influencing MPC cultures, represents the mayor limit of this study. Serum is highly complex biological fluid composed also by many other macromolecules (i.e., cytokines and immunoglobulins), different lipids, hormones and other small molecules. Further investigations are needed to exhaustively define the essential components required to produce high quality MPC-based cell product. Anyway, here we clearly demonstrate that managing the interactions of these hGFs with their receptors could also represents a new tool in MSC production. In fact, culturing bone marrow in AS, or PhABS, and in presence of *Erlotinib*-HCl and *Nintedanib* could lead to highly purified MPCs that could act as culture initiating cells to produce more homogeneous and synchronized MSC cultures. Those cultures could be easily obtained applying the same HS, by removing receptor inhibition and supplementing with recombinant hGF cocktail including at least EGF and FGF-2, as demonstrated here. Our findings are in accordance with Yamaguchi et al. that reported comparable results between FBS and PhABS, in MSC culture, when PhABS cultures had been supplemented with 10 ng/ml of FGF-2 (Yamaguchi et al., 2002). However, the extensive literature on serum supplements in MSC cultures is very controversial. A number of authors showed no significant differences between AS and FBS (Yamamoto et al., 2003; Spees et al., 2004; Stute et al., 2004), while Shigeno and Ashton obtained greater response using AS (Shigeno and Ashton, 1995), in contrast to the extensively reported higher performances applying FBS (Koller et al., 1998; Kuznetsov et al., 2000). It is reasonable hypothesized that the origins of those controversial data, in MSC manufacturing, reside in the lack of preliminary characterization of hGF content in the HS applied. We also believe that those inconsistencies originate from restricting focus on MSCs, without taking into account the positive effects of the MPC presence within the CBMP once implanted *in vivo*.

HS batches rich in EGF and FGF-2 apparently worsen the production of standard monomorphic MPC cultures probably supporting the proliferation of MSC-like cells from other distinct and rare progenitors reported in the bone marrow. These mesengenic progenitors could include skeletal stem cells (SSCs), recently described as the genuine skeletal tissue stem cells (Bianco and Robey, 2015) or CD146-positive non-stem osteoprogenitor (Sacchetti et al., 2007). This hypothesis is validated by our data on growth factor receptor expression. Significantly higher levels of *EGFR, FGFR2*, and *PDGFRA* were reported in MSCs suggesting that these specific growth factors could have trophic effect on MSC-like cells (Ng et al., 2008) instead of inducing MPCs to differentiate. Conversely, VEGF-A was detected at higher concentrations in MSC-inducing sera while *KDR* was more expressed in MPCs, together with *TGFBR2*, suggesting a role for VEGF-A and possibly TGF-β in MPC differentiation toward the mesenchymal lineage. IGF-1 appeared to be acting as MPC-promoting factor as its levels were particularly high in MPC-inducing sera LZM, LZMF, and SERL, mirroring IGF receptor expression in MPCs.

In conclusion, we believe that MPCs represent also a promising alternative to hBM-MNCs as culture-initiating cells in the production of clinical grade MSCs. A purified and well-characterized progenitor cell population cultured under specific controlled conditions would significantly improve CBMP reproducibility and consequently the predictability of pre-clinical studies. MPCs are found at frequencies one to two logs higher than the other MSC progenitors described in bone marrow. Moreover, future clinical applications for CBMPs based on undifferentiated and not expanded MPCs could take advantage from their angiogenic potential that is suggestive of possible beneficial effects on neo- or re-vascularization of target tissues. A complete definition of the active growth factor cocktail for MPC efficient isolation, expansion, and differentiation is still required also in order to possibly develop specific chemically defined media (CDM), which will eliminate the biological variability related to the serum supplementation.

## Author contributions

MM: Conception and design, Data collection, assembly, analysis, and interpretation. SB: Data collection, assembly, analysis, and interpretation. VC: Collection and assembly of RT-PCR data. FF: Collection and assembly of ELISA assay data. FP: Data collection. IP: Conception and design. SP: Conception and design; Data collection, assembly, analysis, and interpretation; Manuscript writing.

## Funding

This work was funded by “Centro per l'Uso Clinico delle Cellule Staminali” (CUCCS) as part of the project “Impiego di cellule stromali mesenchimali di origine midollare nelle pseudoatrosi, cisti ossee di astragalo e osteotomie in plus delle ossa lunghe” (project number 539999_2014_Petrini_CUCCS).

### Conflict of interest statement

The authors declare that the research was conducted in the absence of any commercial or financial relationships that could be construed as a potential conflict of interest.
