# Supplementary material for: Growth Factor Content in Human Sera Affects the Isolation of Mesangiogenic Progenitor Cells (MPCs) from Human Bone Marrow
Source: Front Cell Dev Biol. 2016 Oct 17;4:114. doi: 10.3389/fcell.2016.00114 (PMC5065953; doi:10.3389/fcell.2016.00114)
Supplement: Supplementary file 1 [file Table1.DOCX]

Supplementary Material

**Growth Factor Contents in Human Sera Affects the Isolation of *Mesangiogenic Progenitor Cell*s (MPCs) from Human Bone Marrow**

**Marina Montali, Serena Barachini, Francesca M. Panvini, Vittoria Carnicelli, Franca Fulceri, Mario Petrini and Simone Pacini***

*** Correspondence: Simone Pacini**: simone.pacini@do.unipi.it

# Supplementary Table S1

| **Gene** | **NCBI#** | **Forward Primer** | **Reverse Primer** | **Amplicon** |
| --- | --- | --- | --- | --- |
| *BMPR1A* | NM_004329 | GATGGCTCGTCGTTGTATCAC | AGTCTGGAGGCTGGATTGTG | 219bp |
| *BMPR2* | NM_001204 | GCAGGTTCTCGTGTCTAGG | AATCATCATAAGTTCAGCCATCC | 175bp |
| *EGFR* | NM_005228 | CGTACCAGATGGATGTGAAC | ACCAATACCTATTCCGTTACAC | 209bp |
| *FGFR1* | NM_023110 | CCTCTATGTGGGCATGGTTT | TACAGGAAGGACGATCTGGG | 128 bp |
| *FGFR2* | NM_000141 | AGCCAACCTCTCGAACAGTAT | ACACTGCCGTTTATGTGTGGA | 140 bp |
| *FGFR3* | NM_000142 | CTGGTGTCCTGTGCCTAC | GCCGTTGGTTGTCTTCTTG | 183 bp |
| *IGF1R* | NM_000875 | GAAGGAGGAGGCTGAATAC | GGTCGGTGATGTTGTAGG | 167bp |
| *IGF2R* | NM_000876 | TGGTGCCTACTTGGTGGATG | AACGCCTGGTGTCCTCTTAC | 150bp |
| *KDR* | NM_002253 | CCGCAGAGTGAGGAAGGAG | CCGTAGGATGATGACAAGAAGTAG | 190bp |
| *PDGFRA* | NM_006206 | CGCTGACAGTGGCTACATC | CTTCAATGGTCTCGTCCTCTC | 167bp |
| *PDGFRB* | NM_002609 | AGTCCTGCCTGTCCTTCTAC | GGTGTCCTTGCTGCTGATG | 156bp |
| *TGFBR1* | NM_004612 | TTCGTGGTTCCGTGAGGCAGAG | GACCGCTCGCCGTGGACAG | 218bp |
| *TGFBR2* | NM_003242 | GCTCCAATATCCTCGTGAAGAAC | CCCACCTGCCCACTGTTAG | 115bp |
| *RPL13A* | NM_012423 | CCTGGAGGAGAAGAGGAAAGAGA | TTGAGGACCTCTGTGTATTTGTCAA | 126bp |
| *ACTB* | NM_001101 | CGCCGCCAGCTCACCATG | CACGATGGAGGGGAAGACGG | 120bp |

**
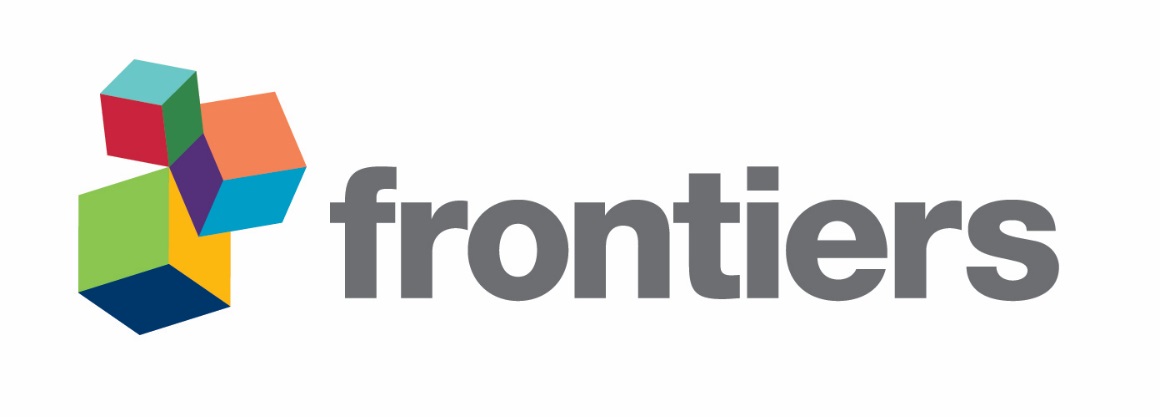
**

**Supplementary Table S1.** Sequences of primer pairs applied in qRT-PCR.
